# The importance of new technologies based on the Internet of Things in reducing drowning 

**Published:** 2022-12

**Authors:** Mostafa Golshekan

**Affiliations:** ^*a*^ Guilan Road Trauma Research Center, Guilan University of Medical Sciences, Rasht, Iran.

## Abstract

**Background:**

The Internet of Things (IoT) is one of the most important and widely used technologies in the world. It has improved processes, reduced costs, and increased safety, and productivity. The use of the IoT in the control of electronic equipment, industrial factories, farm centers, and agricultural fields has been very successful leading to an increase in the amount of production and improved quality of products. Drowning is one of the most important issues in safety and health topics, and many studies and technologies have been designed to reduce the death rate caused by drowning.

**Methods:**

Using the IoT in drowning by designing sensors and creating a communication platform-based internet in swimming pools, entertainment centers, ships, submarines, etc. through increasing the control and accuracy of safety systems can significantly reduce deaths due to drowning. This technology is important in the following aspects of drowning:

i) It improves the performance of safety systems (increasing accuracy and ease of prevention)

ii) It reduces the costs (material and spiritual, such as reducing mortality)

iii) Creating income for knowledge-based companies (creating a market and producing new equipment)

This study was based on the national drowning registry system of Guilan Road Trauma Research Center, Guilan University of Medical Sciences, Rasht, Iran.

**Results:**

IoT in drowning refers to the development and use of IoT in drowning prevention equipment and programs. With a strong focus on machine-to-machine communication, big data, and machine learning, it enables control centers to achieve better efficiency and reliability. By using this technology, you can get more accurate information about air quality, water quality, risks, and climate changes to prevent drowning. IoT devices in this application typically span a large geographic area and can also be mobile.

**Conclusions:**

The development of IoT-based equipment in the marine transportation system, seashores, swimming pools, etc., especially in developing countries, can reduce drowning-related deaths.

**Keywords:**

Drowning, Technology, Internet of Things (IoT), Guilan

